# The WellNext Scan: Validity evidence of a new team-based tool to map and support physicians’ well-being in the clinical working context

**DOI:** 10.1371/journal.pone.0319038

**Published:** 2025-02-26

**Authors:** Sofiya Abedali, Joost van den Berg, Alina Smirnova, Maarten Debets, Rosa Bogerd, Kiki Lombarts

**Affiliations:** 1 Department of Medical Psychology, Research group Professional Performance and Compassionate Care, Amsterdam UMC location University of Amsterdam, Amsterdam, The Netherlands; 2 Amsterdam Public Health Research Institute, Amsterdam, The Netherlands; 3 Department of Internal Medicine and Geriatrics, Amsterdam UMC location University of Amsterdam, Amsterdam, The Netherlands; 4 Department of Family Medicine, University of Calgary, Calgary, Canada; Case Western Reserve University, UNITED STATES OF AMERICA

## Abstract

Occupational well-being is inherent to physicians’ professional performance and is indispensable for a cost-effective, robust healthcare system and excellent patient outcomes. Increasing numbers of physicians with symptoms of burnout, depression, and other health issues are demonstrating the need to foster and maintain physicians’ well-being. Assessing physicians’ well-being, occupational demands, and resources can help create more supportive and health-promoting working environments. The WellNext Scan (WNS) is a 46-item questionnaire developed to assess (i) physicians’ well-being and (ii) relevant factors related to physicians’ clinical working environment. We collected data to investigate the validity and reliability of the WNS using a non-randomized, multicenter, cross-sectional survey of 467 physicians (staff, residents, doctors not in training, and fellows) from 17 departments in academic and non-academic teaching medical centers in the Netherlands. Exploratory factor analysis detected three composite scales of well-being (energy and work enjoyment, meaning, and patient-related disengagement) and five explanatory factors (supportive team culture, efficiency of practice, job control and team-based well-being practices, resilience, and self-kindness). Pearson’s correlations, item-total and inter-scale correlations, and Cronbach’s alphas demonstrated good construct validity and internal consistency reliability of the scales (*α*: 0.67–0.90; item-total correlations: 0.33–0.84; inter-scale correlations: 0.19–0.62). Overall, the WNS appears to yield reliable and valid data and is now available as a supportive tool for meaningful team-based conversations aimed at improving physician well-being.

## Introduction

Physician well-being is inherent to effective professional performance and is indispensable for a cost-effective, robust healthcare system and excellent patient outcomes. [1–5] However, physicians worldwide are encountering threats to their well-being, as evidenced by widespread reports of burnout, symptoms of depression, and other health issues. [6–12] Individual characteristics such as resilience and self-compassion can also contribute to well-being, while low self-compassion and low resilience are linked to a higher risk of burnout symptoms. [13–15]

Although various initiatives have been launched to enhance physicians’ well-being, the focus has leaned towards individual approaches to enhancing well-being. [13–17] However, the focus on individual approaches does not sufficiently address essential organizational cultural elements, such as shared ways of thinking, ideas, and behaviors within the working unit [18,19] and structural elements in the organization, including systems used to support and enable good workflow and performance. Moreover, research shows that focusing only on individual approaches without aiming to change professional values or organizational culture can cause physicians to resist well-being interventions. [20] The vital role of colleagues in physicians’ daily professional lives also needs to be acknowledged, as colleagues are essential factors in well-being through the quality of working relationships, social capital and support, and collective decision-making. [11,21] Following the job demands and resources (JD-R) model, one could describe the cultural and structural determinants of physicians’ working environment as job demands and job resources [22–24], and an imbalance, i.e., high work demands and insufficient job resources, would elevate the risk for diminished well-being. [1,25–28] A health-promoting and supportive culture in the team and the organization could be perceived as a job resource that benefits the well-being of the individual physician or the team. In contrast, research has shown that demanding aspects of the clinical working environment, such as reduced autonomy, high workloads, administrative burdens, and lack of leadership and support, are associated with physician burnout. [7,18,29] These job demands and resources should be addressed on a team- or organizational-level to cultivate a holistic and effective response to physicians’ multifaceted challenges. [17,23,25,30,31]

Research has suggested that the first step in enhancing well-being and creating healthier work environments is to assess and discuss how physicians experience well-being and its underlying influential factors in their daily practice. [16] Discussing shared demands and resources can be a starting point for improving well-being and adapting the working environment by relating to the needs of all team members.

Various valid questionnaires exist for assessing aspects of physicians’ well-being, [32] identifying burnout or distress, [33,34] or evaluating organizational demands and resources affecting occupational well-being. [35] Three issues remain under-addressed in these measures. First, the predominant focus on individual assessments rather than team measures provides limited information and may cause resistance and decrease the effect of following well-being interventions. [20] Second, many current instruments do not incorporate positive and negative well-being indicators, although a more holistic measurement of well-being can be beneficial in designing targeted interventions. [36–38] Third, they are not specific to the influencing factors within the working environment of hospital-based physicians in the Netherlands. Therefore, we aimed to develop a new assessment tool, the WellNext Scan (WNS), that is practice-oriented, team-centered, and user-friendly and reflects a comprehensive approach toward well-being by including important influencing factors in the working environment of physicians. The WNS inquires about (i) elements of organizational culture and climate (i.e., psychological safety, team cohesion, and perceived support for self-care), [39–41] (ii) factors from the organizational structural context that reflect how supported and enabled physicians feel in their work by the department and institution (i.e., efficiency, career development opportunities, autonomy, and daily work hassles), and lastly incorporates items relating to individual strengths and resources (resilience, self-kindness, and self-care). [18,29,42,43] With this approach, the WNS recognizes the interconnected nature of individual well-being and broader team and organizational influences.

In this paper, we elaborate on the WNS development and aim to investigate 1) the validity and 2) the reliability of the WNS questionnaire. The aim of developing the WNS and its use in practice is to facilitate meaningful physician team dialogues to address and foster physician well-being in the workplace. Utilizing the WNS provides physician teams with tailor-made reports to guide collaborative reflection and prioritize potential improvement actions.

## Materials and methods

### WellNext scan questionnaire development

#### Rationale of measuring well-being.

The concept of well-being has been variably defined in the past decades. However, many definitions include descriptions of its multidimensional nature, which encompasses both positive and negative aspects. [32,42–48] Using the eudaimonic and hedonic well-being strands in philosophy, well-being can be described as an affective state of emotional, physical, mental, and social nature. Eudaimonic well-being refers to living a purposeful and meaningful life with continued personal growth and quality ties to others. Hedonic well-being describes what makes experience and work life (un)pleasant based on an individual evaluation – such as happiness and satisfaction. [43,49–51] Likely, an individual who functions well in life will also have positive feelings about this life. [50,52,53] The combination of both well-being philosophies has been proposed before as research suggests that they are interconnected, and both philosophical perspectives provide a more comprehensive understanding of well-being. [54–57] The importance of including positive and negative well-being indicators to provide a more holistic understanding of the dynamic interactions of well-being facets has also been stressed in research before. [36,58] Positive and negative experiences are not merely opposites of each other but are rather distinct dimensions that could contribute to well-being independently. [36–38] Including positive and negative well-being indicators also has the potential to allow for designing targeted interventions, preventative measures, and adequate support to enhance positive well-being and mitigate negative well-being by linking specific and common factors in the working context that contribute to positive or negative well-being experiences. This can be useful in identifying root causes for positive affect or distress. [36,37,58,59]

#### Background and aims of instrument development.

The WNS was built upon a previously developed prototype tool that resulted from a larger well-being intervention by Debets et al. [60] amongst 377 physicians from 48 teams in multiple Dutch hospitals. The project included a literature review, a needs assessment, a 75-item questionnaire assessing well-being and working conditions, the design of a feedback report, and guided follow-up amongst four teams. [60] Physicians’ positive response to the piloted well-being intervention led to the decision to develop a short, practice-focused tool that is easily accessible and facilitates a meaningful discussion by providing teams with a team-level report.

The WNS was designed for hospital-based teams with physicians across roles (faculty, staff, residents, junior doctors, and fellows) to evaluate team members’ experienced well-being, the associated elements of organizational culture and structure within their teams and organizations, and physicians’ psychological strengths. The rationale of the research team for the new instrument prescribed that the WNS would (i) acknowledge the multidimensional nature of well-being and its dependence on individual and organizational factors, (ii) acknowledge measures deemed relevant by physicians as determined in the previous project, (iii) make use of validated constructs when available and (iv) acknowledge the potential administrative burden for its users by limiting each influencing domain to a maximum of 10 items, while considering that it can be filled out in maximum 10 minutes.

#### Instrument development.

We used multiple approaches to generate items describing the factors influencing physician well-being within the clinical working context. The identified influencing factors were initially grouped into (i) elements of organizational culture and climate, (ii) organizational structural context, and (iii) individual strengths and resources, resembling the three proposed domains of the Stanford model of professional fulfillment framework, that suggests that the culture at work, the efficient organization of practice, and physicians’ resilience are interconnected and affect physicians’ professional fulfilment. [18,61]

The primary information source for the influencing factors was the pilot of the preceding well-being program, described in more detail in the study of Debets et al. [60] Next, KL, MD, and RB updated the literature search (unpublished material), extracted the most relevant factors linked to physicians’ well-being and burnout on individual, team- and organizational levels and sought validated scales for the respective items. [16,30,62,63] KL, MD, and RB selected ten items for the individual, cultural, and structural factors, respectively. We adapted items from existing questionnaires to better fit the context and objectives of the WNS and added items that reflect specific work-related topics and norms. The domain elements of organizational culture and climate will be used as an example. Here, we combined items that reflect psychological safety culture and psychological safety climate with items on team cohesion for a holistic view of the support that physicians experience from their close colleagues and department leadership. The S1 File , Theoretical Background of the WellNext Scan, lists all items and their theoretical origins. [18,61] MD, KL, and RB adapted items to the Dutch language and healthcare context and their cultural relevance to our target population, i.e., Dutch physicians in different training and practice levels working in teams with multiple other physicians.

The final selection of items was presented to physicians independent of the research team regarding the clarity and relevance of the items for studying physicians’ well-being, and significant factors in their working environment, that describe elements of organizational culture and structure. [64] With the feedback from these physicians, we adjusted the WNS questionnaire, which was subsequently piloted.

#### Measures.

The final WNS questionnaire comprises 46 items, leaving room for free text comments after each section. The response categories for all subscales of the WNS were unified to a 5-point Likert scale, ranging from 1 =  “Not at all true” to 5 =  “Completely true.” Three items were phrased negatively (see Table 3). All definitions of the WNS sub-scales and their measures are explained in more detail in the S1 File, Theoretical Background of the WellNext Scan.

#### Well-being.

To measure well-being in the WNS, we used a validated measure that assesses physicians’ professional fulfillment, work exhaustion, and interpersonal disengagement that captures both hedonic (i.e., “I feel happy at work”) and eudaimonic (i.e., “My work is meaningful to me”) aspects of physician occupational well-being is the Professional Fulfilment Index (PFI). The PFI has a multidimensional view of well-being, including physical, emotional, mental, and social dimensions. It is measured with professional fulfillment (n = 6), work exhaustion (n = 4), and interpersonal disengagement related to patient care (n = 6). All items are originally scored on a 5-point Likert scale (0–4). The cutoff scores are 3 for professional fulfillment and 1.33 for work exhaustion and interpersonal disengagement and were calculated by averaging all subscale items. The cutoff scores for the Dutch context have not been calculated yet, but a high score on professional fulfillment is positive, and, in contrast, a low score on work exhaustion and interpersonal disengagement can be considered positive.

#### Influencing factors.

The *organizational culture and climate* factor elements were measured with items reflecting psychological safety (climate), perceived possibilities of part-time work, and team cohesion. [39,41] The *organizational structural context* was measured with career development opportunities, [65] autonomy, and perception of supportive workplace systems. *Individual strengths and resources* reflect the measures for resilience, [66] self-kindness, [67] and self-care strategies, such as daily balance and cognitive awareness. [68] The theoretical links of these concepts to well-being are further elaborated in S1 File.

### WellNext scan questionnaire validation

#### Study setting.

This study was a non-randomized, cross-sectional survey of physician teams consisting of junior doctors, residents, fellows, and staff from multiple (non)-academic medical centers in the Netherlands. In the Netherlands, recent medical graduates can work as junior doctors before residency training or enter residency training directly from medical school. Residency training, lasting up to six years, is a prerequisite for becoming a registered medical specialist. In the Netherlands, there are regulations, mandates and initiatives promoting the well-being of healthcare professionals, including physicians. Specifically, any medical specialist working with residents and junior doctors is expected to provide supervision and education and must, thus, comply with Dutch well-being mandates, according to which resident well-being may be considered part of the quality assessment of residency training programs. [33] These mandates may include working hours regulations, work-life balance provisions, and mental health and well-being support systems. Thus, junior doctors, residents, fellows, and staff collectively contribute to the well-being and influencing factors in the working environment. Furthermore, Dutch residents are increasingly involved in departmental decisions, including decisions about their training program and how healthcare is delivered in their department. This is partly because regularly discussing clinical and teaching performance is common and partially integrated into physicians’ training and re-registration regulations. As a result of recent mandates, monitoring the well-being of physicians has become part of the physician teams’ quality improvement cycle, which is usually initiated by the organization or department. Departments generally have access to multiple tools and interventions including interventional groups, coaches, mindfulness, and resilience trainings, but most interventions target the individual.

#### Data collection.

Members of physician teams, including faculty, residents, junior doctors, and fellows working in academic and non-academic teaching hospitals in the Netherlands, were invited to participate. Data were collected between March 2021 and July 2023 using a nationally known web-based data collection platform to support regular quality assurance and improvement activities in medical education and professional development. Invitations and several reminders were sent based on the preferences of the local initiator of the WNS, usually departmental leadership or program director. Participation was not mandatory, but team members were encouraged to participate. Participants could fill out the questionnaire at any given moment within the set time frame after receiving their invitation and fill out the questionnaire individually. It was not possible to skip questions on the WNS items, and partial questionnaires were not included in the analysis. Thus, this study has no missing data on the WNS items. However, there is missing demographic information, for example, regarding gender. Participants provided written consent for their data to be used for scientific research on the WellNext Scan before starting the survey. The WNS findings were summarized in team reports and fed back to the participating teams anonymously. For the WNS reports, measures were installed to safeguard and protect physician anonymity.

### Statistical analysis

Before conducting the primary analyses, we screened the data using descriptive statistics on the demographic variables of participants’ age (below 25 to above 70 with increments of 5 years), gender (female/male/other), function (medical specialist/ resident/ junior doctor/ fellow), type of hospital (academic hospitals/ specialty clinics/ general hospitals/ top clinical hospitals), and specialty (medical/ surgical/ non-medical/ supportive). Medical specialties were categorized as shown in Table 1 below.

**Table 1:** Categorization of medical specialties.

Medical	Surgical
Internal medicine	Ophthalmology
Cardiology	Orthopedics
Pediatrics	Urology
Pulmonary diseases	Oral surgery
Gastrointestinal and liver diseases	Cardio-thoracic surgery
Neurology	
Rehabilitation medicine	**Non-medical**
Psychiatry	Medical Psychology
SEH	Pharmacy—Pharmacy
Dermatology	Clinical Physics
Intensive care	Clinical Chemistry
ICL	Mental Health
General practitioners	Medical Microbiology and Immunology
Nursing home physicians	Sexology
Sports medicine	Special Dentistry
Clinical geriatrics	Clinical Neurophysiology
Internal medicine	Dentistry
**Surgical**	**Supporting**
Surgery	Anesthesiology
Gynecology	Clinical Genetics
ENT	Pathology
Neurosurgery	Radiology
Plastic surgery	Radiotherapy

Further, we addressed and identified measurement error outliers by exploring the data and its normality with Kolmogorov-Smirnov tests, which utilize Lilliefors’ significance correction.

To investigate the validity and reliability of the WNS, we conducted analyses concerning construct validity and internal consistency reliability. Construct validity refers to the ability of a test instrument to capture and measure an abstract (theoretical) concept. Ideally, this process is based on multiple sources of information. [69,70] Reliability relates to the consistency of responses across different scales, indicating how consistent the instrument measurement is. [71] Testing reliability supports the interpretation of whether the items within each factor consistently measure the same underlying construct. [72] To examine the construct validity of the WNS, we examined the structure of the well-being constructs separately from the influencing factor constructs using exploratory factor analyses.

#### Physician well-being.

For the well-being construct, we first examined the original factor structure of the PFI with confirmatory factor analysis (CFA), as it is an already validated measure. We tested a model with three correlating factors (professional fulfillment, work exhaustion, and interpersonal disengagement) with robust maximum likelihood estimation. The model fit was assessed with the Comparative Fit Index (CFI) and the Tucker-Lewis Index (TLI) (>0.95 good fit; ≥  0.90 acceptable fit), the Root Mean Squared Error of Approximation (RMSEA) (<0.06 good fit; < 0.10 poor but acceptable fit), and the x^2^/ df ratio < 5 rule. [73–75] We screened for multicollinearity and tested multiple models, with and without including weaker loading items for each subscale of the PFI. However, the model fit was poor on all indicators (see Appendix, CFA results), which suggested that the PFI model did not fit the data of our study population. Thus, we consequently performed an EFA for all 16 PFI items using principal component analysis with oblimin rotation as used in the original validation study of the PFI. The strategic shift to an EFA aligns with recommended practices in multivariate analysis and instrument development to ensure the model is theoretically sound and empirically validated. [76] The CFA was conducted using the program R, version 4.2.1, with the package *lavaan*. All other analyses were performed using SPSS, version 26 (IBM et al., 2012).

#### Influencing factors.

To explore the structure of the influencing factors – theoretically categorized in elements of organizational culture and climate, organizational structural context, and individual strengths and resources—we first conducted sample adequacy tests (Kaiser-Mayer-Olkin measure (>0.6 acceptable), Bartlett’s test of sphericity (p < 0.005)) to assess the significance of all correlations and factorability and sample adequacy of the data for the EFA. [77–80] The minimal sample size compliant with the criteria for EFA (between 7–10 cases per variable) suggested in the literature for construct validation and scale refinement was reached with our sample size of 467 participants. [71] Then, we utilized an exploratory factor analysis (EFA) using principal axis factoring with oblique rotation, as the factors were expected to correlate. [71]

For the EFA’s on the well-being construct and the influencing factors, items were assessed for sufficient contribution to the factor model by analyzing communalities (>0.2). Factor structure was determined by analyzing factor loadings (>0.3), the ratio of cross-loadings of items on multiple factors (<75%), the scree plots, and the Kaiser criterion (Eigenvalue > 1). [71] Only items that did not meet these criteria were removed from further analyses.

#### Reliability and internal consistency.

To assess the internal consistency reliability of all composite scales yielded by the factor analyses and to examine how well individual items correlate with their respective scales and with items in other scales, Cronbach’s α (α=0.60 moderate; α=0.80 strong), item-total, and inter-scale correlations were calculated. Pearson’s correlations with 95% confidence intervals (CI) were used to determine the strength of the item-total and inter-scale correlations. Item-total scale correlations of 0.40 or higher were considered acceptable evidence of the contribution of each item to the scale homogeneity. Inter-scale correlations for the well-being scales and the well-being influencing scales were utilized separately were used to check for the interpretability of the composite scales as distinct albeit correlated constructs (for correlations =< 0.70) (r < 0.30 negligible, r =  0.30 – 0.49 weak, r =  0.50 – 0.70 moderate, and r >  0.71 strong).

### Ethical review

The study received a waiver from the Institutional Ethical Review Board of the Amsterdam Medical Centers (reference number W23_124 # 23.151). The waiver concerns ethical aspects, data management, and privacy issues, including general data protection regulation (GDPR).

## Results

### Participants

The final sample included 467 participants from 17 participating teams from multiple medical specialties. Table 2 shows the descriptive statistics of the study population. The participating teams were diverse in composition, ranging in size from three to fifty members, and included residents, junior doctors, fellows, and staff, usually from a single medical discipline per team.

**Table 2:** Descriptive statistics of the study population.

Baseline characteristic	Category	N = 467	
		*n*	%
Gender	Female	173	37
	Male	94	20.2
	Other	–	–
Valid N		267	57.2
Missing N		200	42.8
Age	≤=25	9	1.9
	26–30	49	10.5
	31–35	37	7.9
	36–40	41	8.8
	41–45	35	7.5
	46–50	20	4.3
	51–55	19	4.1
	56–60	23	4.9
	61–65	–	–
	66–70	3	0.6
	>70	1	0.2
Vaid N		237	50.7
Missing N		230	49.3
Type of hospital	Academic	274	58.7
	Specialty Clinic	81	17.3
	General	53	11.3
	Top clinical	28	6
Valid N		436	93.4
Missing N		31	6.6
Specialty	Medical	320	68.5
	Surgical	144	26.9
	Non-medical	3	0.6
	Supportive	–	–
Valid N		467	100
Missing N			
Function	Medical Specialists	279	59.7
	Residents	141	30.2
	Junior doctors	36	7.7
	Fellows	11	2.4
Valid N		467	100
Missing N			

**Table 3:** Results from exploratory factor analysis of the well-being subscales in the WNS questionnaire.

WNS subscales and items (variance in %), α*	*Mean*	*Factor loading* ^#^	*h*^2^*	*Item-total correlation* ^&^
*Factor 1: Energy and work enjoyment N**** = ****9 (43.36%), α****=*** *0.88* *Indicate the extent to which the following statements apply to your work experience in the past two weeks.*	*3.78*			
EW 1. I feel happy at work.	*3.72*	*0.70*	*0.70*	*0.69*
EW 2. I feel worthwhile at work.	*3.60*	*0.69*	*0.53*	*0.57*
EW 3. I feel in control when dealing with difficult problems at work. *In the past two weeks, have you felt:*	*3.51*	‒*0.48*	*0.49*	*0.55*
EW 4. (R) A sense of dread when I think about work that I have to do.	*3.62*	*0.62*	*0.52*	*0.63*
EW 5. (R) Physically exhausted at work.	*3.73*	*0.72*	*0.55*	*0.63*
EW 6. (R) Lacking enthusiasm at work.	*3.94*	*0.65*	*0.64*	*0.72*
EW 7. (R) Emotionally exhausted at work.	*3.87*	*0.77*	*0.64*	*0.72*
EW 8. (R) Less empathetic with my colleagues.	*4.09*	*0.61*	*0.64*	*0.53*
EW 9. (R) Less connected with my colleagues.	*4.00*	*0.69*	*0.60*	*0.61*
*Factor 2: Meaning* *N**** = ****3 (13.86%), α****=*** *0.76* *Indicate the extent to which the following statements apply to your work experience in the past two weeks.*	*4.0*			
M 1. My work is satisfying to me.	*3.97*	*0.65*	*0.73*	*0.62*
M 2. My work is meaningful to me.	*4.19*	*0.75*	*0.65*	*0.61*
M 3. I am contributing professionally in the ways I value most (e.g., patient care, teaching, research, and leadership).	*3.85*	*0.64*	*0.53*	*0.53*
*Factor 3: Patient-related disengagement* *N**** = ****4 (7.10%), α****=*** *0.90* *In the past two weeks, have you felt:*	*4.29*			
PD 1. (R) Less empathetic with my patients.	*4.30*	‒*0.86*	*0.76*	*0.79*
PD 2. (R) Less sensitive to others’ feelings/emotions.	*4.18*	‒*0.72*	*0.69*	*0.67*
PD 3. (R) Less interested in talking with my patients.	*4.34*	‒*0.90*	*0.82*	*0.84*
PD 4. (R) Less connected with my patients.	*4.34*	‒*0.89*	*0.79*	*0.81*

*Note. N* =  467. The extraction method was principal axis factoring with an oblique (oblimin, delta = 0 with Kaiser Normalization) rotation. Reverse-scored items for the analysis are denoted with an (R). α*= Internal consistency reliability coefficient Cronbach’s alpha (α=0.60 moderate; α=0.80 strong). *h*^2*^=communalities. # = factor loading on primary scale. & = corrected item-total correlations.

### Sample data characteristics.

There were no missing data values for the WNS items. The data was not normally distributed (Kolmogorov-Smirnov Normality Test, p = 0.001). Bartlett’s test of sphericity was significant for the EFA on the well-being constructs (χ2 (120) =  4288.45, p < 0.000) and the influencing factors (χ2 (378) =  3674.24, p < 0.000), showing that the chosen analytic model was appropriate for the given data. The Kaiser-Meyer-Olkin measure of sampling adequacy was high for all scales (KMO: Well-being: 0.90; EFA on influencing factors: 0.87). [81]

### The WellNext Scan structure: construct validity, reliability, and internal consistency.

#### Confirmatory factor analysis on the well-being constructs of the professional fulfillment index.

The original structure of the professional fulfillment index was not confirmed using a robust maximum likelihood confirmatory factor analysis by specifying one model with three correlating scales. The model was significant (Chi^2^ =  p = 0.00) but suggested a poor fit on all used indicators (CFI = 0.86; TLI = 0.83; RMSEA = 0.11).

In a second model, when analyzing the three subscales separately with a robust maximum likelihood confirmatory factor analysis, professional fulfilment and work exhaustion suggested an acceptable to good model fit according to the CFI (PF: 0.958; WE: 0.995) but an unacceptable fit for interpersonal disengagement (0.852). The Tucker-Lewis-Index only showed an acceptable fit for the subscale work exhaustion (WE: 0.985) but not for professional fulfillment and interpersonal disengagement (PF: 0.935; ID = 0.753). Finally, RMSEA was acceptable for professional fulfillment and work exhaustion but not for ID (PF: 0.103; WE: 0.061; ID: 0.261). The models were significant (x^2^ =  0.00)

#### Physician well-being.

The exploratory factor analysis on the well-being construct showed a new structure of three composite sub-scales, which we labeled ‘energy and work enjoyment’ (n = 9), ‘meaning’ (n = 3), and ‘patient-related disengagement’ (n = 4). Table 3 reports the factor loadings, explained variance, communalities, reliability coefficients (Cronbach’s alpha), and the corrected item-total correlations for the three identified well-being subscales. *Energy and work enjoyment* capture happiness, physical and emotional exhaustion, connectedness, and empathy toward colleagues. *Meaning* refers to a sense of purpose and value alignment with work, and *patient-related disengagement* captures aspects specifically related to patients. The factor loadings for all scales were above 0.60, except for one item, ‘I feel in control when dealing with difficult problems at work’ (part of the *energy and work enjoyment* scale), which still loaded at 0.48. Cronbach’s alphas were high for all three composite scales, varying from α =  0.76 for *meaning* to α =  0.90 for *patient-related disengagement*. The item-total correlations were above 0.50 for all items within their respective scales and varied between 0.53 and 0.84. The explained variance for the extracted three scales was satisfactory, and the communalities for each item were acceptable to good (range 0.49 - 0.82).

The inter-scale correlations were below the targeted 0.70 threshold and ranged from 0.34 between meaning and patient-related disengagement to 0.62 between energy and work enjoyment and meaning. Pearson’s correlations between the well-being constructs (Table 4) were significant with the strongest correlation between meaning and energy and work enjoyment (0.62), and the lowest between patient-related disengagement and meaning (0.36).

**Table 4:** Pearson’s correlations of the WNS well-being scales.

	Energy and work enjoyment		Meaning	Patient-related disengagement
Energy and work enjoyment		1	.62**	.54**
Meaning		.62**	1	.36**
Patient-related disengagement		.54**	.36**	1

N=467.

**. Correlation is significant at the 0.01 level (2-tailed).

#### Influencing factors.

**Fig 1:**
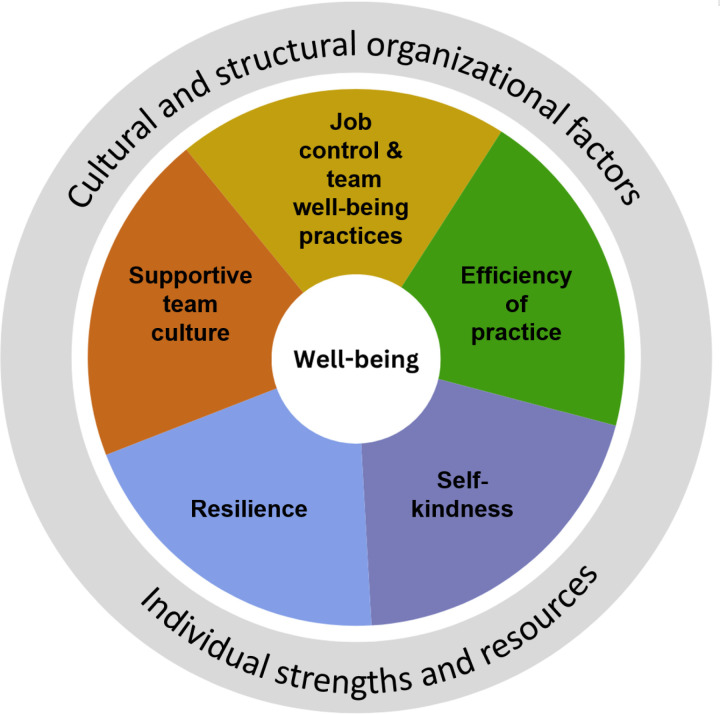
The WellNext Scan structure. Well-being and influencing factors in the working environment.

Table 5 gives an overview of the psychometrics of the influencing factor scales resulting from the EFA. The EFA on the 30 items revealed five influencing factors consisting of four to seven items per scale. Three factors described organizational aspects of the working environment: (1) ‘supportive team culture’ ‘ (n=6), (2) ‘job control and team-based well-being practices’ (n = 7), and (3) ‘efficiency of practice’ (n=5). Two factors related to individual physicians’ psychological coping ability are (4) ‘resilience’ (n = 4) and (5) ‘self-kindness’ (n = 6) (see Fig 1). Two items, “I experience a lot of administrative burden in my work” from the factor describing the organizational structural context and “Working part-time is considered quite normal in our department,” did not fit any scale and were therefore excluded from further analyses but will be retained as independent items in the WNS as they are informative for practice.

**Table 5:** Results from exploratory factor analysis of the influencing factors subscales in the WNS questionnaire.

Influencing domains (extracted variance in %), α *	*Mean*	*Factor loading* ^#^	*h*^2^*	*Item-total correlation* ^&^
**Factor 1: Supportive team culture (4.61%),** *α= 0.75*	**3.74**			
1. I can be myself when working with my close colleagues.	4.02	*0.59*	0.47	0.57
2. It is possible to address problems and difficult issues in our department.	*3.52*	*0.51*	0.41	0.51
3. In our department, colleagues take care of each other	3.70	*0.54*	0.45	0.55
4. (R) Colleagues sometimes reject others because they are different.	3.75	0.52	0.24	0.39
5. I am comfortable discussing my well-being with colleagues.	3.56	0.60	0.51	0.59
6. (R) It is difficult to ask colleagues for help in our department.	3.93	0.35	0.19	0.40
**Factor 2: Job control and well-being practices (21.95%),** *α****=*** *0.79*	**3.29**			
1. Well-being is regularly on the agenda in our department.	2.91	0.36	0.32	0.45
2. My unique skills and talents are valued and utilized in our department.	3.53	0.46	0.51	0.58
3. The department management takes the well-being of colleagues seriously.	3.54	0.57	0.47	0.59
4. I am involved in important departmental decisions.	3.07	0.63	0.44	0.57
5. I have a satisfactory say in the scheduling process.	3.48	0.65	0.44	0.57
6. I can determine myself how much time I spend with a patient.	2.78	0.46	0.24	0.39
7. The department offers me sufficient learning and development opportunities.	3.76	0.50	0.37	0.52
**Factor 3: Efficiency of practice (6.20%),** *α****=*** *0.69*	**3.45**			
1. The systems in our department are in the service of providing good patient care.	3.35	0.71	0.48	0.51
2. The patient is our department’s highest priority.	3.85	0.64	0.39	0.49
3. The purpose of the departmental tasks is usually clear.	3.73	0.50	0.38	0.48
4. Work in our department is done efficiently.	3.12	0.51	0.37	0.48
5. I am adequately supported if I have ICT problems during my work.	3.21	0.35	0.14	0.32
**Factor 4: Resilience (5.38%),** *α****=*** *0.67*	**4.03**			
1. I am able to adapt to change.	4.03	0.71	0.54	0.58
2. I make a proactive effort to manage the challenges of my professional work.	3.93	0.72	0.55	0.53
3. I tend to bounce back after illness or adversity.	4.16	0.36	0.27	0.40
4. I monitor my feelings and reactions to patients.	4.03	0.41	0.19	0.33
**Factor 5: Self-kindness (8.01%),** *α****=*** *0.74*	**3.16**			
1. I take some time for relaxation each day.	3.22	0.40	0.26	0.42
2. I’m kind to myself when I’m experiencing suffering.	2.97	0.73	0.58	0.59
3. I try to be understanding and patient toward those aspects of my personality I do not like.	3.32	0.61	0.43	0.51
4. I avoid over-commitment to work responsibilities.	2.82	0.53	0.27	0.41
5. I am mindful of triggers that increase professional stress.	3.46	0.38	0.24	0.41

*Note. N* =  467. The extraction method was principal axis factoring with an oblique (oblimin, delta = 0 with Kaiser Normalization) rotation. Reverse-scored items for the analysis are denoted with an (R). α*= Internal consistency reliability coefficient Cronbach’s alpha (α=0.60 moderate; α=0.80 strong). *h*^2*^=communalities. # factor loading on primary scale. & = corrected item-total correlations. All questions in the influencing domains had the following prompt: “Please indicate the extent to which you agree or disagree with the following statements.”

The factor loadings were acceptable to good and ranged between 0.40 and 0.73 for all scales, with one item in each factor loading between 0.30 and 0.40. Cronbach’s alphas were moderate to good for all five composite scales, varying from α =  0.67 for resilience to α =  0.79 for job control and team-based practices. The item-total correlations for all items within their respective scales ranged from 0.33, ‘I am adequately supported if I have ICT problems during my work’ (factor 3), to 0.59, for the item, ‘I am comfortable discussing my well-being with colleagues’ (factor 1). The explained variance for the extracted five scales was good, and the communalities for most retained items sufficiently contributed to the factor model (commonalities 0.14 – 0.58). The inter-scale correlations (Table 6) were below the targeted 0.70 threshold. They ranged from 0.19 between self-kindness and efficiency of practice and 0.53 between supportive team culture, and job control and team-based well-being practices.

**Table 6:** Pearson’s correlations of the WNS influencing factors.

	Supportive team culture	Job control and team-based well-being practices	Efficiency of practice	Resilience	Self-kindness
Supportive team culture	1	.53**	.34**	.41**	.21**
Job control and team-based well-being practices	.53**	1	.41**	.37**	.27**
Efficiency of practice	.34**	.41**	1	.29**	.19**
Resilience	.41**	.37**	.29**	1	.33**
Self-kindness	.21**	.27**	.19**	.33**	1

N=467.

**. Correlation is significant at the 0.01 level (2-tailed).

## Discussion

### Main findings

This study introduces a new practice-oriented team-based tool – the WellNext Scan—to help map the well-being of physicians working in hospitals and the individual and organizational factors that influence their well-being. The findings suggest that physicians’ well-being can be captured and reliably measured by three correlating scales: energy and work enjoyment, meaning, and patient-related disengagement. Additionally, we identified five influencing factors related to individual (resilience, self-kindness) and organizational (supportive team culture, efficiency of practice, and job control and team-based well-being practices) characteristics. All scales showed good construct validity and moderate to strong internal consistency reliability.

### Explanation of results

#### Physician well-being.

To have a meaningful team dialogue about well-being, insight into how team members experience their well-being in the working context may be a helpful start. To measure physicians’ well-being, as part of the WNS, we used the three-factor professional fulfillment index developed by Trockel et al. (2018), previously validated in the US context. Our exploratory factor analysis demonstrated three factors but with differently composed scales compared to previous PFI research [31,63,82,83], underpinning the importance of additional validation of instruments when used in new contexts. Two things stand out when comparing the original PFI and the well-being scales we identified. First, where the original professional fulfillment (PF) scale combines eudaimonic well-being aspects (i.e., experiencing meaning in work) and hedonic aspects of well-being (i.e., being happy), Dutch physicians view these two as different. We extracted a robust, separate new scale, which we labeled *meaning,* representing the three eudemonic items referring to fulfillment, meaning, and recognition of one’s values in work. The other three PF items were grouped with those measuring work exhaustion as part of a new scale measuring *energy and work enjoyment*. Second, the original PFI contains the subscale Interpersonal Disengagement, which includes items referring to relationships with colleagues and patients. Again, Dutch physicians have a different view: items relating to patients clustered strongly as a separate new scale, named *patient-related (dis)engagement*, whereas the colleagues-related items were part of the newly identified *energy and work enjoyment* scale. This finding resonates with previous studies, showing that physicians might absorb joy and anger from their colleagues more strongly than from their patients through emotional contagion. [84] Colleagues can be perceived as psychosocial job resources (or demands) – contributing to energy and job satisfaction – potentially moderating the relationship between workload and well-being. [85] A study with Dutch physicians showed that whereas patients observed a professional attitude, colleagues were able to detect changes in physicians’ psychological states, such as high or low work engagement. [86] Similarly, our results mirror previous findings that, on the one hand, emotionally demanding interactions with patients are experienced as a typical job demand of physicians. However, on the other hand, physicians can also derive energy from patient interaction as they are essential for the meaning and fulfillment related to their work. [1,87,88]

Although the well-being constructs’ structure differed in our sample, the analyses showed that three distinct well-being constructs best fit the data. The factor loadings, communalities, and the corrected item-total correlations of each item in the subscales were good according to the selection criteria previously explained. Cronbach’s alphas also demonstrated that the internal consistency of the new subscales is reliable, and Pearson’s correlations indicated that the constructs are correlated but distinct. Professional fulfillment may be perceived differently across cultures due to variations in healthcare organizational structures and cultures, policies, and practices. The linguistic adaptation of the PFI could also have affected physicians’ interpretation of the PFI items.

#### Influencing factors.

To measure influencing factors in the working context of physicians, we grouped multiple previously validated scales into three overarching factors: 1) elements of organizational culture and climate, 2) organizational structural context, and 3) individual strengths and resources. However, our analysis revealed five factors in total. The individual strengths and resources factor components are subdivided into two scales: *resilience* and *self-kindness*. Items from the self-care scale addressing cognitive strategies loaded with resilience, and items relating to daily balance loaded with self-kindness, showing the importance of recognizing and addressing early signs of stress, fatigue, and irritability for both resilience and self-kindness. [68] This clustering resonates with previous research, as resilience and self-kindness are separate important predictors of physicians’ well-being and performance that stimulate professional fulfillment [14,32,89,90], and self-care strategies are hypothesized to promote resilience and self-kindness. [68] Previous research has shown that self-kindness, mindfulness, and self-care actions, reflected in the items of the *self-kindness* scale, are often employed as coping strategies by physicians. [8,13–15] A recent study in the Netherlands showed that self-kind physicians were more fulfilled because they were more resilient and could balance work with private life. [91]

Next, elements of organizational culture and organizational structural context items were grouped into three scales representing organizational aspects differently than we had constructed them. From the items in the theoretically designed structural factor, efficiency and supportive workplace systems comprised the new factor of *efficiency of practice*. The remaining items of this initial factor—autonomy and career development opportunities—grouped with items representing organizational culture that measure psychological safety, perceived appreciation of one’s skills, and departmental well-being practices in the factor we named *job control and team-based well-being practices*. This new clustering reflects the views on practicing physicians in the Netherlands in hospital-based contexts compared to the initial theoretical structure. It provides additional validity evidence for the WNS as a new tool for use in clinical practice. The results also highlight the need to investigate the validity and reliability of the new tools. Researchers may start from an organizational knowledge base, approaching a work environment in terms of its organizational culture and structure, as done in this study. However, the results exposed how physicians view their work contexts. Through their eyes, the new factor of *job control and team-based well-being practices* indicates perceived leadership trust in prioritizing well-being, employee growth, and autonomy on a departmental level. In previous studies, all named concepts show positive associations with professional well-being and satisfaction among physicians [29,30,92–94]. This new factor combines aspects relating to self-perception of person-organization fit and how enabled physicians feel to function and thrive at their job, which is in line with previous research. [88,95,96]

Lastly, the remaining items in the theory-based factor measuring elements of organizational culture (psychological safety, psychological safety climate, and team cohesion) composed the new factor *supportive team culture*, which enquires about perceptions of workplace consequences tied to interpersonal risk-taking, mutual support, inclusivity, trust, care, and open communication among colleagues. [41,97,98]

#### Implications for clinical practice and medical education.

Using the WNS as a general scan of physicians’ well-being in the occupational context may be instrumental to starting an open dialogue about well-being in teams. It may be supportive in getting to know team members’ perspectives on well-being and work contexts, raising awareness about (un)healthy behaviors or practices, or monitoring well-being improvement actions. In the Netherlands, a recent national study amongst physicians showed severe threats to social safety in clinical work environments, with more than half of the participants reporting having experienced a form of intimidation, sexual harassment, bullying, or discrimination. [99] Professional societies or healthcare institutions could introduce or suggest the WNS for their members or teams to start conversations about social safety at work. We recommend that experienced professionals facilitate these conversations, as this could benefit the understanding, acceptance, and integration of the feedback, often available in Dutch hospitals through human resources or the medical education departments. [41,100–102] The WNS offers a valuable, comprehensive tool for physician teams to assess and address these concerns, providing insights into the culture within their working teams and guiding efforts to promote well-being. The rich information provided by the WNS can be used to elucidate points for improvement and help create a meaningful conversation about the results in the department. These points for improvement are deducted from factors or specific items within the WNS report that score very low or high on a team level. We recommend that teams discuss the results of the WNS with a professional coach or facilitator, who will help team members to find solutions together and aim to include all team members in the discussion. This meaningful conversation, in which points for improvement and maintenance are found together as well as solutions, is an important step in creating a better working environment as a team. [97,103]

Teams participating in the WNS can receive anonymized feedback reports with the summarized data of their evaluation. Within Dutch healthcare settings, hierarchical structures often exist, with specific team members holding more authority or decision-making power than others. [104] These power differences may affect how respondents perceive and report on the working culture in daily work, potentially leading to underreporting negative experiences or hesitancy in expressing dissenting opinions. [104,105] The WNS and its report serve as valuable tools in overcoming the challenges posed by potential formal and informal power structures within the team. By providing a platform for all team members to share their insights and suggestions in an anonymized report, the WNS aims to foster inclusivity and equal participation. This approach ensures that strengths and areas for improvement within the working environment are sourced from diverse perspectives, contributing to a more comprehensive understanding of the team dynamics, and facilitating collaborative efforts towards positive change. Repeated measurement and regular discussion of the measurement results are recommended to ensure continuous improvement. We suggest a two-step approach to discussing the WNS report in physician teams. At first, residents and faculty meet separately, guided by a facilitator, to discuss the results and topics raised in the WNS. Afterward, assisted by a facilitator, both groups collectively analyze the main points and formulate conclusions and suggestions for follow-up actions. After 6 to 12 months, the WNS evaluation is repeated, and previously formulated goals are reviewed.

For medical education, a better understanding of positive and negative well-being and its determining factors in the work context of residents can inform well-being interventions and the design of residency training curricula that are better aligned with the well-being needs of residents. By providing information on the workplace, the residents, and the clinical supervisors, the WNS can elucidate points for improvement and assist leadership in providing tailored guidance in maintaining well-being, for example, through coaching. [16,106]

Dutch hospitals’ organizational structures and healthcare practices may not directly translate to other healthcare systems. Factors such as healthcare financing, governance models, and cultural norms within the Dutch context could influence the applicability of WNS findings in diverse international settings as they impact organizational structures, practices, and workforce dynamics.

Additionally, the relatively open view around well-being and performance measures in the Dutch medical culture must be accounted for when considering how the WNS might perform in other cultures and organizational contexts, as cultural norms, hierarchy structures, and organizational aspects can affect how physicians prioritize and interpret the items and the usefulness of the WellNext Scan. In the Netherlands, well-being is likely more openly discussed than in other countries, as it is a formal part of the quality assessment of residency training. Although they are limited, available structures from previously implemented policies can be used for follow-ups of the WNS measurement, such as available coaches that can facilitate the discussion of the results.

The influencing domains of the WNS were designed by incorporating elements that are broadly applicable across various healthcare contexts, however, their interpretation and impact might differ for different physician groups or practice settings. Cross-cultural validations should consider assessing how social and cultural elements, and organizational norms impact the interpretation and relevance of WNS items. [64]

#### Implications for research and policy.

Physician well-being is not solely an individual responsibility but is also linked to key roles outlined in the CanMEDS framework, a competency framework widely adopted in the Netherlands to maintain professional standards. [107,108] The “Professional” role asks physicians to maintain their health to uphold high standards of professionalism, recognizing the interdependence between personal well-being and optimal patient care. By adopting a comprehensive approach to addressing well-being, such as utilized in the WNS, physicians may be better able to comply with the requirement of self-care in the professional roles of the CanMEDS and share this responsibility with other healthcare professionals in their teams.

This study has multiple implications for research. In this initial phase in the validation process of the WNS, we established the factor structure of well-being and a range of its predictors in the working environment. Kane [69] refers to this type of validity evidence as the ‘scoring argument,’ one of four necessary first steps to take in any systematic instrument development and validation approach. Validation of any socially embedded instrument, such as the WNS, is continuous, and repeated validation over time is recommended. To further advance the robustness of the WNS, future research could continue the validation process of the WNS regarding the subsequent inferences of the argument in Kane’s validity framework, including generalization, extrapolation, and, ultimately, implications of using the WNS. [70] This could include the social impact and consequences on physicians’ well-being and team follow-up decisions based on their usage of the WNS.

While physicians’ well-being and many influencing factors in the working context represent common resources and demands for physicians internationally, cross-cultural adaptations of the WNS are necessary to account for unique characteristics and (national and inter-organizational) cultural and systemic variations in healthcare that affect organizational support towards physicians’ well-being, and social support and cohesion in the team. [64] Future research could also investigate whether the structure of the WNS varies for different contexts within healthcare (private practices, non-teaching medical centers) or for different respondent groups, i.e., female, male, and gender-diverse residents versus faculty. Additionally, a specialty-specific use of the WNS can be explored. [109–111] With larger sample sizes, a generalizability analysis could estimate the minimum number of questionnaires needed for the sub-scale scores on the team level and confirm the structure of the questionnaire using confirmatory factor analysis. [74] Given the poor fit of the original PFI model resulting from the CFA, it is possible that the differences in the characteristics of our sample population, i.e., diversity in training levels and specialties, introduced a variability that the initial model did not capture. The different structure of the well-being constructs in our study compared to the original PFI highlights the need for future studies to investigate whether the newly defined structure is also robust on a faculty-only sample and when including residents, fellows, or other (non) hospital-based medical professionals.

#### Strengths and limitations of this study.

The study’s strengths include utilizing the multifaceted view of positive and negative well-being with the hedonic and eudaimonic well-being perspectives. [50,55,56,112] Second, this study was conducted in multiple (non-)academic teaching medical centers in the Netherlands with a physician population from various specialties, supporting and strengthening the results’ generalizability.

This study has two main limitations. The current sample size allowed us to conduct an EFA to investigate the construct validity of the WNS but not a CFA to evaluate the construct validity, which is considered ideal for instrument validation. [74,113] Further, the perception of the measure as potentially mandatory or linked to negative repercussions might have contributed to an increased social desirability bias, particularly as initiating the WNS measure was typically driven by team leadership. It is possible that teams with proactive well-being-oriented leadership were more likely to engage with the WNS, potentially overlooking teams with less emphasis on well-being in their leadership. Therefore, a CFA on a larger sample that includes a higher variability in team leadership styles would be desired.

## Conclusion

To support physicians’ well-being and professional performance in contemporary medical practice, the WellNext Scan provides a team-based instrument that informs about well-being and its influencing factors in the work context. The results suggest that the WellNext Scan can validly and reliably inform physician teams about their well-being and its associated determinants within the occupational context. Clinical teams may use the WNS as a first step in discussing and enhancing their well-being. Future research must continue to validate the WNS, for example, by demonstrating improvements in well-being and working conditions over time. Lastly, cross-cultural comparative studies can help in understanding how perceptions of well-being and professional fulfillment and the impact of factors in the working environment vary across different cultures and healthcare systems.

## Supporting information

S1 File Supplementary material. Theoretical background of the WellNext Scan.
(DOCX)
