# Supplementary material for: The WellNext Scan: Validity evidence of a new team-based tool to map and support physicians’ well-being in the clinical working context
Source: PLoS One. 2025 Feb 26;20(2):e0319038. doi: 10.1371/journal.pone.0319038 (PMC11864550; doi:10.1371/journal.pone.0319038)
Supplement: S1 File — Theoretical background of the WellNext Scan. (DOCX) [file pone.0319038.s001.docx]

## Supplementary file 1

## 1. WellNext Scan

## Well-being

Table 1, Theoretical background and operationalization of WNS sub-scales

| **Construct** | **Item** | **Question/Statement** | **Background** |
| --- | --- | --- | --- |
| Professional fulfillment | 1 | I feel happy at work. | Operationalization:  To assess vitality, we used the 16-item professional fulfillment index (PFI), developed and validated for US physicians.[1] The PFI consists of three subscales i) professional fulfillment (6 items), ii) work exhaustion (4 items) and iii) interpersonal disengagement (6 items).[1]  A higher score on the professional fulfilment scale is favorable while a high score on the work exhaustion and interpersonal disengagement scales suggests a higher likelihood of burnout or unwellness. |
|  | 2 | I feel worthwhile at work. |  |
|  | 3 | My work is satisfying to me. |  |
|  | 4 | I feel in control when dealing with difficult problems at work. |  |
|  | 5 | My work is meaningful to me. |  |
|  | 6 | I’m contributing professionally in the ways I value most (e.g. patient care, teaching, research, and leadership). |  |
| Work exhaustion | 7 | A sense of dread when I think about work I have to do. | Theoretical Background:  Professional fulfillment (PF) relates to intrinsic need satisfaction, which is positively associated to professional performance and engagement. [1] PF includes intrinsic positive reward derived from work and contains “dimensions of happiness, self-worth, self-efficacy, and satisfaction at work” [1] but also the two burnout measures work exhaustion and interpersonal disengagement. PF includes positive and negative indicators of well-being, and relates to both, the eudaimonic characteristics and actions, such as self-realization (self-worth and self-efficacy) and needs fulfillment, and the hedonic qualities of vitality – an individual evaluation of (work) life – such as happiness and satisfaction with the job or profession. [2] [3] Insights on professional fulfillment have shown that while positive factors influencing the intrinsic motivation are often related to the work itself, the de-motivators are often linked to extrinsic aspects such as administrative burdens, autonomy, work environment or interpersonal relationships at work. For physicians’ occupational context, the PFI provides additional information that is valuable for practice, as it is capturing interpersonal disengagement relating to patient care, by assessing empathy and connectedness towards patients and colleagues. |
|  | 8 | Physically exhausted at work. |  |
|  | 9 | Lacking in enthusiasm at work. |  |
|  | 10 | Emotionally exhausted at work. |  |
| Interpersonal disengagement | 11 | Less empathetic with my patients. |  |
|  | 12 | Less empathetic with my colleagues. |  |
|  | 13 | Less sensitive to others’ feelings/emotions. |  |
|  | 14 | Less interested in talking with my patients. |  |
|  | 15 | Less connected with my patients. |  |
|  | 16 | Less connected with my colleagues. |  |

## Elements of Organizational Culture and Climate

The domain organizational culture and climate comprises values, knowledge, skills, attitudes, shared ways of thinking, ideas and behaviors that are valued within a healthcare organization or social group (the department or organization) [4] This composite measure shows the perceived support for self-care, professional and personal development, and empathy for one's coworkers, patients, and oneself. [4, 5] Elements of organizational culture and climate describes employees’ perceptions and experiences of their work environment and shared values, norms, and practices that shape behaviour within the organization. Shared norms and practices can be understood as accepted ways of behaving and interacting within the team, including communication styles, teamwork, and support. The domain also describes aspects of the organizational climate, for example how employees perceive the level of support and resources available for their professional development and well-being. A higher score reflects a more supportive cultural context.

| **Construct** | **Item** | **Question/Statement** | **Background** |
| --- | --- | --- | --- |
| Team cohesion | 1 | I can be myself when working with my close colleagues. | Operationalization:  Team cohesion items were self-formulated. |
|  | 4 | In our department, colleagues take care of each other. |  |
| Psychological Safety Climate [6] (rephrased) | 2 | Well-being is regularly on the agenda in our department. | Operationalization: Psychological safety climate was operationalized with rephrased items from the psychological safety climate scale. [6]  Theoretical Background:  The psychological safety climate (PSC) scale by Hall et al., 2013 is a component of organizational climate and is defined as “shared perceptions regarding policies, practices, and procedures reflected in a communicated organizational position concerning the value of the psychosocial health and safety of employees in the workplace”. [6]The PSC measurements indicate whether leaders and managers in the organization value employee’s psychosocial health. The psychological safety climate can be seen as a macro-level job resource and has been examined to have a moderating effect on job distress. Psychological safety climate specifically is a job resource that can buffer the effects of job demands on depression and moderate between depression and engagement and job satisfaction. 58 60 In a study among direct support professionals, depression was associated with work overload, and was especially dependent on the availability of organizational resources such as decision-making related to staff, material supplies, and overall funding. [7] High levels of PSC are therefore associated with reduced job demands and increased job resources, as it was found to be “negatively related to increases in work pressure and emotional job demands and positively related to decreases in both psychological distress, and emotional exhaustion over time” [8], as well as with increased work engagement through the positive relationship with resources. [6] |
|  | 6 | I am comfortable discussing my well-being with colleagues. |  |
|  | 10 | The department management takes the well-being of colleagues seriously |  |
| Psychological safety [9] | 3 | It is possible to address problems and difficult issues in our department. | Operationalization:  Psychological safety was operationalized with items evaluating psychological safety from the psychological safety scale. [9]  Theoretical Background:  Psychological safety refers to the perceptions people have of the consequences of taking interpersonal risks in the workplace. [9] In the context of organizational culture, high psychological safety has been linked to reduced job demands, such as work pressure and emotional job demands and increased job resources, such as decreases in psychological distress and emotional exhaustion.[8] Medical teams can decrease detrimental stress by ensuring that all team members feel comfortable addressing difficulties without fear of retaliation or other negative reactions from peers or superiors. [5]  Psychological safety in the occupational setting of physicians has been shown as an important predictor for professional development, proactive learning, and knowledge sharing [10]. The lack of psychological safety can hinder team collaboration and decrease safety performance among healthcare staff. [11, 12] Paying attention to psychologically safe working environments for physicians is necessary, as it is an essential part of a supportive team culture linked to well-being, with the potential to improve patient safety and professional development of the individual physicians and the team. [13] |
|  | 5 | Colleagues sometimes reject others because they are different. |  |
|  | 7 | My unique skills and talents are valued and utilized in our department. |  |
|  | 9 | It is difficult to ask colleagues for help in our department. |  |
| Well-being policy | 8 | Working part-time is considered quite normal in our department. | This question is derived from practice and addresses the possibility and perception of part-time work. The question addresses flexibility at work and is an indicator for possibilities of work-life balance in the department. |

## Organizational Context

The Organizational Context domain assesses critical aspects influencing the work environment for physicians in a healthcare organization. It focuses on efficiency, career development opportunities, autonomy, and daily work hassles, providing insights into how these factors impact the overall well-being and professional experience of healthcare professionals. A higher score reflects a more efficient and enabling working environment.

| **Item** | **Item** | **Question/Statement** | **Underlying construct** |
| --- | --- | --- | --- |
| Supportive workplace systems | 1 | The systems in our department are in the service of providing good patient care. | The questions regarding supportive workplace systems for practice efficiency were formulated by the research team based on practical needs of physicians in practice. |
|  | 6 | I am adequately supported if I have ICT problems during my work. |  |
| Efficiency [14] | 2 | The patient is our department's highest priority. | Operationalization:  Efficiency and the priority of tasks was operationalized with items from the questionnaire on the experience and evaluation at work (QEEW), which were validated as part of the QEEW. [14] |
|  | 3 | The purpose of the departmental tasks is usually clear. |  |
|  | 4 | Work in our department is done efficiently. |  |
| Hassle at work | 5 | I experience a lot of administrative burden in my work. | Operationalization:  This question was derived from practice but was also a reoccurring factor mentioned in the international scientific literature on physician well-being. It is related to experiencing hassle at work or distress because of administrative tasks. High administrative burden can have a negative impact on physicians’ ability to provide good quality patient care and has been linked to lower career satisfaction and increased risk for burnout in physicians. [15] |
| Autonomy | 7 | I am involved in important departmental decisions. | Operationalization: These questions were formulated by the research team to specifically address autonomy related to the clinical working environment.  Theoretical Background: Following the self-determination theory (SDT), developed by Deci and Ryan, [16] autonomy is identified as a fundamental psychological need that is crucial for motivation and well-being. For physicians, autonomy can involve having control over clinical decisions and aligning their work with personal values and professional standards. SDT posits that autonomy is essential for both hedonic (pleasure-based) and eudaimonic (meaning-based) well-being. [17] When physicians feel autonomous, they are more likely to experience job satisfaction, reduced burnout, and greater psychological resilience. Research supports this, showing that autonomy-supportive environments enhance psychological health by fulfilling basic psychological needs. For instance, previous studies found that autonomy is linked to greater well-being and reduced stress and indicate that those who have more control over their work schedules and decision-making processes report higher job satisfaction and lower levels of burnout. Thus, better understanding and fostering autonomy in the medical profession is key to promoting well-being, enabling physicians to provide better patient care while maintaining their own mental health and professional fulfillment. [17-21] |
|  | 8 | I have a satisfactory say in the scheduling process. |  |
|  | 9 | I can determine myself how much time I spend with a patient. |  |
| Career development [14] | 10 | The department offers me sufficient learning and development opportunities. | Operationalization:  Career development opportunities was operationalized with one item from the questionnaire on the experience and evaluation at work (QEEW). It was validated as part of the QEEW. [14] |

## Individual Strengths and Resources

A higher score reflects the ability to react to stressful situations, to be kind to oneself and to detect when stress is occurring and implement self-care practices.

| **Construct** | **Item** | **Question/Statement** | **Background** |
| --- | --- | --- | --- |
| Resilience [22] | 1 | I am able to adapt to change. | Operationalization:  Resilience was operationalized with the CD-RISC2 scale, [22] which was validated for use with patients in the USA. [22]  Theoretical Background:  *Personal resilience* refers to personal qualities that enable one to thrive in the face of adversity, the ability to react to a stressful emotional situation in a healthy manner and to “bounce back”. [22]  Resilience is one of the central components of (physician’s) occupational well-being [23] and could be seen as a measure of successful coping ability. [24] |
|  | 6 | I tend to bounce back after illness or adversity. |  |
| Self-care: *cognitive strategies [25]* | 2 | I make a proactive effort to manage the challenges of my professional work. | Operationalization:  *Self-care* was measured with questions from the modules “daily balance” and “cognitive awareness” of the Professional Self-Care Scale (PSCS). [25] The PSCS was validated for use with psychologists. [25]  Theoretical Background:  *Self-care* is assumed as a “means of avoiding the adverse effects of stress and promoting professional functioning and well-being”. [25] |
|  | 8 | I am mindful of triggers that increase professional stress |  |
|  | 9 | I monitor my feelings and reaction to patients. |  |
| Self-care: *daily balance [25]* | 3 | I take some time for relaxation each day. |  |
|  | 7 | I avoid over-commitment to work responsibilities. |  |
| Self-kindness [26] | 4 | I’m kind to myself when I’m experiencing suffering. | Operationalization:  *Self-kindness* was operationalized with the self-kindness module from the validated self-compassion scale by Neff. [26]  Theoretical Background:  *Self-compassion* has an important role in the development of human potential [27] and is defined as the “acceptance of human experience vs. the pursuit of perfect medical practice” and is composed of three reciprocal components: 1. self-kindness, 2. feelings of common humanity, and 3. mindfulness. Self-kindness specifically refers to how individuals react to own shortcomings and own self-care. Thus, a self-kind attitude would be opposite to a self-judging attitude; it is a type of self-acceptance that decreases the feelings of unworthiness. [26, 28]  “Also, having compassion for oneself implies that individuals will try to prevent the experience of suffering in the first place, giving rise to proactive behaviors aimed at promoting or maintaining well-being (e.g., taking time off from work before becoming overly stressed).” [28]  Self-kindness, specifically “involves more than ending self-criticism, however. It involves actively showing concern for our distress.” [28] Research has also shown that self-compassion is a positive predictor of well-being and contributes to greater emotional resilience. [28] |
|  | 5 | I try to be understanding and patient toward those aspects of my personality I do not like. |  |
|  | 10 | I am tolerant of my own flaws and inadequacies. |  |

**References**

1. Trockel, M., et al., *A brief instrument to assess both burnout and professional fulfillment in physicians: reliability and validity, including correlation with self-reported medical errors, in a sample of resident and practicing physicians.* Academic Psychiatry, 2018. **42**(1): p. 11-24.

2. Bartels, A.L., S.J. Peterson, and C.S. Reina, *Understanding well-being at work: Development and validation of the eudaimonic workplace well-being scale.* PloS one, 2019. **14**(4): p. e0215957.

3. Ryan, R.M. and E.L. Deci, *On happiness and human potentials: A review of research on hedonic and eudaimonic well-being.* Annual review of psychology, 2001. **52**: p. 141.

4. Mannion, R. and H. Davies, *Understanding organisational culture for healthcare quality improvement.* Bmj, 2018. **363**.

5. Bohman, B., et al., *Physician well-being: the reciprocity of practice efficiency, culture of wellness, and personal resilience.* NEJM Catalyst, 2017. **3**(4).

6. Hall, G.B., et al., *Psychosocial safety climate buffers effects of job demands on depression and positive organizational behaviors.* Anxiety, Stress, & Coping, 2013. **26**(4): p. 355-377.

7. Gray-Stanley, J.A., et al., *Work stress and depression among direct support professionals: the role of work support and locus of control.* Journal of Intellectual Disability Research, 2010. **54**(8): p. 749-761.

8. Dollard, M.F. and A.B. Bakker, *Psychosocial safety climate as a precursor to conducive work environments, psychological health problems, and employee engagement.* Journal of occupational and organizational psychology, 2010. **83**(3): p. 579-599.

9. Edmondson, A., *Psychological Safety and Learning Behavior in Work Teams.* Administrative Science Quarterly, 1999. **44**(2): p. 350-383.

10. Scheepers, R.A., et al., *Physicians' Perceptions of Psychological Safety and Peer Performance Feedback.* J Contin Educ Health Prof, 2018. **38**(4): p. 250-254.

11. Singer, S., et al., *Relationship of safety climate and safety performance in hospitals.* Health services research, 2009. **44**(2p1): p. 399-421.

12. Edmondson, A.C. and K.S. Roloff, *Overcoming barriers to collaboration: Psychological safety and learning in diverse teams*, in *Team effectiveness in complex organizations*. 2008, Routledge. p. 217-242.

13. Hall, J., et al., *The Clinical Learning Environment in CanMEDS 2025.* Can Med Educ J, 2023. **14**(1): p. 41-45.

14. Veldhoven, M., J. Prins, P. van der Laken, and L. Dijkstra, *VBBA2.0: Update van de standaard voor vragenlijstonderzoek naar werk, welbevinden en prestaties*. 2014.

15. Rao, S.K., et al., *The Impact of Administrative Burden on Academic Physicians: Results of a Hospital-Wide Physician Survey.* Acad Med, 2017. **92**(2): p. 237-243.

16. Ryan, R.M., V. Huta, and E.L. Deci, *Living well: a self-determination theory perspective on eudaimonia.* Journal of Happiness Studies, 2008. **9**(1): p. 139-170.

17. Babenko, O., *Professional Well-Being of Practicing Physicians: The Roles of Autonomy, Competence, and Relatedness.* Healthcare, 2018. **6**(1): p. 12.

18. Clausen, T., et al., *Job autonomy and psychological well-being: A linear or a non-linear association?* European Journal of Work and Organizational Psychology, 2022. **31**(3): p. 395-405.

19. Fotiadis, A., K. Abdulrahman, and A. Spyridou, *The Mediating Roles of Psychological Autonomy, Competence and Relatedness on Work-Life Balance and Well-Being.* Frontiers in Psychology, 2019. **10**.

20. Olson, K., et al., *Organizational strategies to reduce physician burnout and improve professional fulfillment.* Curr Probl Pediatr Adolesc Health Care, 2019. **49**(12): p. 100664.

21. Tummers, L., B. Steijn, B. Nevicka, and M. Heerema, *The effects of leadership and job autonomy on vitality: Survey and experimental evidence.* Review of public personnel administration, 2018. **38**(3): p. 355-377.

22. Vaishnavi, S., K. Connor, and J.R. Davidson, *An abbreviated version of the Connor-Davidson Resilience Scale (CD-RISC), the CD-RISC2: psychometric properties and applications in psychopharmacological trials.* Psychiatry Res, 2007. **152**(2-3): p. 293-7.

23. Zwack, J. and J. Schweitzer, *If every fifth physician is affected by burnout, what about the other four? Resilience strategies of experienced physicians.* Academic Medicine, 2013. **88**(3): p. 382-389.

24. Connor, K.M. and J.R. Davidson, *Development of a new resilience scale: the Connor-Davidson Resilience Scale (CD-RISC).* Depress Anxiety, 2003. **18**(2): p. 76-82.

25. Dorociak, K.E., P.A. Rupert, F.B. Bryant, and E. Zahniser, *Development of the Professional Self-Care Scale.* J Couns Psychol, 2017. **64**(3): p. 325-334.

26. Neff, K.D., *The development and validation of a scale to measure self-compassion.* Self and identity, 2003. **2**(3): p. 223-250.

27. Neff, K.D., *Self‐compassion, self‐esteem, and well‐being.* Social and personality psychology compass, 2011. **5**(1): p. 1-12.

28. Neff, K.D., *Self-Compassion: Theory, Method, Research, and Intervention.* Annual Review of Psychology, 2023. **74**(1): p. 193-218.
